# Reversible Modification of Rashba States in Topological Insulators at Room Temperature by Edge Functionalization

**DOI:** 10.1002/advs.202519814

**Published:** 2025-11-20

**Authors:** Wonhee Ko, Seoung‐Hun Kang, Qiangsheng Lu, An‐Hsi Chen, Gyula Eres, Ho Nyung Lee, Young‐Kyun Kwon, Robert G. Moore, Mina Yoon, Matthew Brahlek

**Affiliations:** ^1^ Department of Physics and Astronomy The University of Tennessee Knoxville Tennessee 37996 USA; ^2^ Materials Science and Technology Division Oak Ridge National Laboratory Oak Ridge Tennessee 37831 USA; ^3^ Department of Information Display Kyung Hee University Seoul 02447 South Korea; ^4^ Department of Physics, and Research Institute for Basic Sciences Kyung Hee University Seoul 02447 South Korea; ^5^ Research Center for Technology Commercialization Korea Institute of Science and Technology Information (KISTI) Seoul 02456 South Korea

**Keywords:** density functional theory, functionalization, Rashba edge states, scanning tunneling microscopy, topological insulators

## Abstract

Quantum materials with novel spin textures from strong spin‐orbit coupling (SOC) are essential components for a wide array of proposed spintronic devices. Topological insulators have a necessary strong SOC that imposes a unique spin texture on topological states and Rashba states that arise on the boundary, but there is no established methodology to control the spin texture reversibly. Here, it is demonstrated that functionalizing Bi_2_Se_3_ films by altering the step‐edge termination directly changes the strength of SOC and thereby modifies the Rashba strength of 1D edge states. Scanning tunneling microscopy/spectroscopy shows that these Rashba edge states arise and subsequently vanish through the Se functionalization and reduction process of the step edges. The observations are corroborated by density functional theory calculations, which show that a subtle chemical change of edge termination fundamentally alters the underlying electronic structure. Importantly, fully reversible and repeatable switching of Rashba edge states across multiple cycles at room temperature is experimentally demonstrated. The results imply Se functionalization as a practical method to control SOC and spin texture of quantum states in topological insulators.

## Graphical abstract

The Rashba edge states at the step edges of Bi_2_Se_3_ display switching behavior, according to the functionalization and defunctionalization of step edges with Se atoms that are achieved by annealing in UHV and Se flux conditions, respectively.

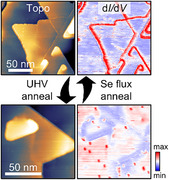

## 1 Introduction

Topological insulators are bulk insulators characterized by an inverted band structure primarily induced by strong spin‐orbit coupling (SOC).^[^ ^1^, ^2^ ^]^ This band inversion establishes a non‐trivial topological order that guarantees the existence of robust surface states protected by time‐reversal symmetry. Moreover, the strong SOC in these materials also leads to the formation of Rashba states at the surface or edges when inversion symmetry is broken.^[^ ^3^, ^4^ ^]^ Both topological surface states and Rashba states exhibit spin texture from spin‐momentum locking, i.e., each momentum state is associated with a unique spin direction. The unique spin texture of these states facilitates efficient conversion between charge and spin, which makes topological insulators promising for spintronic device applications.^[^ ^5^ ^–^ ^12^ ^]^

Although these advantageous properties make topological materials of general interest for spintronic devices, practical applications require control over the spin texture as well as energy scales for which these phenomena can be observed up to ambient conditions near or above room temperature.^[^ ^13^ ^]^ From the atomistic perspective, the intrinsic strength of SOC is determined by elemental composition and lattice geometry, making it difficult to modulate without altering the material itself. Traditional approaches for tuning SOC often involve modifying either the gross material composition of the bulk or its underlying lattice structure,^[^ ^14^ ^]^ which is mostly irreversible. Therefore, finding sensitive tuning “knobs” for the strength of SOC, and thus overall spin texture, that is both reversible and apparent at room temperature is highly desirable for the study of topological insulators as well as future spintronic applications.

Here, we demonstrate that the functionalization of 1D step edges in topological insulators is a highly sensitive platform that enables tuning SOC and thereby switching emergent Rashba edge states reversibly through the edge termination. We investigated 10 quintuple layer (QL) Bi_2_Se_3_ films grown by molecular beam epitaxy (MBE) (see Methods for detailed growth conditions), which is a prototypical topological insulator.^[^ ^15^, ^16^ ^]^ The film thickness of 10 QL was chosen because Rashba edge states only exist for Bi_2_Se_3_ films thicker than the critical thickness of ≈5 QL,^[^ ^17^ ^]^ while the surface uniformity is better for thinner films. Because the films are grown under the excessive Se flux, step edges are terminated by Se atoms and manifest Rashba edge states in scanning tunneling microscopy/spectroscopy (STM/S) study at room temperature as significantly enhanced d*I*/d*V* signal along the step edges.^[^ ^17^, ^18^, ^19^ ^]^ However, further annealing in the ultra‐high vacuum (UHV) environment (<10^−9^ mbar) drives a fundamental change to the edge state as seen in d*I*/d*V* spectroscopy at room temperature, where the signal along the step edges disappears. Subsequent reannealing of the films in Se flux fully recovers the Rashba edge states signal. Repeating these two processes of annealing in UHV and annealing in Se flux suppressed and fully recovered the edge states each time, demonstrating complete reversibility. To elucidate the origin of the switching behavior, we employed tight‐binding models parameterized by density functional theory (DFT) calculations of the step edges with different terminations. The Se‐terminated edges display localized edge states with large Rashba interaction strength, but the Bi‐terminated edges show Rashba edge states pushed into the bulk valence band with significantly reduced Rashba interaction strength. The results experimentally and theoretically demonstrate the reversible and repeatable switching of Rashba edge states by Se functionalization of step edges, which offers a huge advantage over previous non‐reversible methods, such as external doping,^[^ ^4^ ^]^ composition variation,^[^ ^14^ ^]^ and thickness control,^[^ ^17^ ^]^ for realizing devices with topological materials.

## 2 Results and Discussion

**Figure 1:**
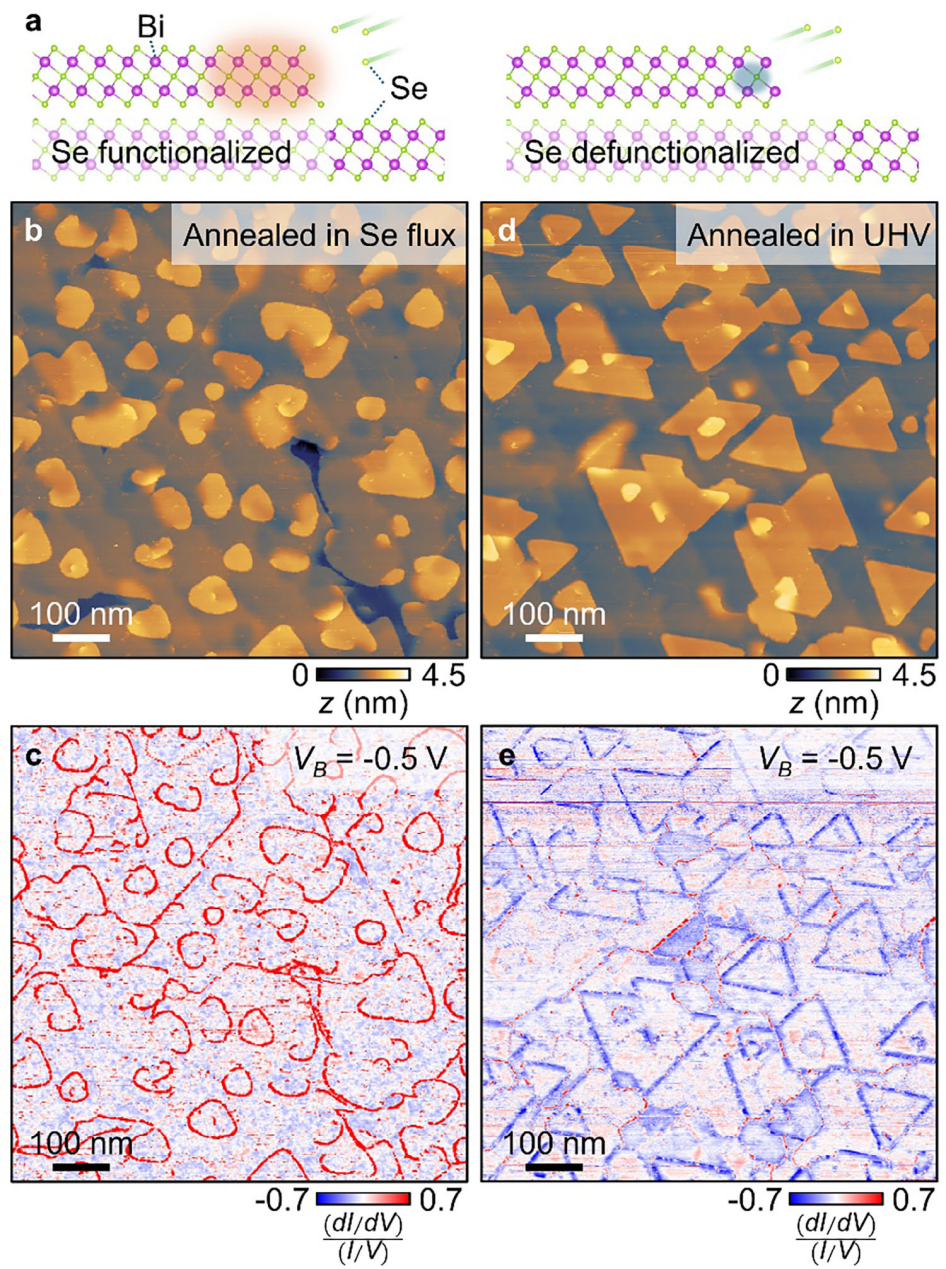
Rashba edge states in Bi_2_Se_3_ depending on functionalization with Se atoms. a) (left) Schematic of the Bi_2_Se_3_ edge functionalization with Se atoms and strongly enhanced Rashba edge states, and (right) schematic of the Bi_2_Se_3_ edge defunctionalization by removing Se atoms and suppressed Rashba edge states. b) Large‐scale topographic images of the 10 QL Bi_2_Se_3_ film after annealing in Se flux (*V_B_* = −0.5 V, *I* = 10 pA). c) Normalized d*I*/d*V* maps at *V_B_* = −0.5 V simultaneously taken with the topographic image in (b). d) Large‐scale topographic images of the 10 QL Bi_2_Se_3_ film after annealing in UHV environment (*V_B_* = −0.5 V, *I* = 5 pA). e) Normalized d*I*/d*V* maps at *V_B_* = −0.5 V simultaneously taken with the topographic image in (d).

**Figure** **1**a shows the schematic of the Bi_2_Se_3_ edge functionalization and defunctionalization, respectively. Here, edge functionalization specifically means forming Se‐terminated step edges, which is experimentally realized either by initially growing Bi_2_Se_3_ films with excessive Se (ten times of Bi flux), or by post‐annealing the films at 200 °C with a comparable amount of Se flux as used during the growth for about an hour. Edge defunctionalization, in opposition, denotes a process of forming Bi‐terminated step edges by detaching Se atoms, which is experimentally achieved by annealing the films at 200 °C with no Se flux in UHV (<10^−9^ mbar) for about an hour. When terminated with Se atoms, the step edges display significantly enhanced d*I*/d*V* signal due to the emergence of Rashba edge states, while desorbing those terminating Se atoms suppresses d*I*/d*V* signal from Rashba edge states. Figure 1b shows a large‐scale topographic image of 10 QL Bi_2_Se_3_ film functionalized by Se atoms. The image displays a large flat terrace with islands mostly 1 QL high (≈1 nm), which indicates high‐quality layer‐by‐layer growth of Bi_2_Se_3_ films by MBE. Figure 1c shows the d*I*/d*V* maps at *V_B_* = −0.5 V taken simultaneously with Figure 1b, which clearly shows the strongly enhanced d*I*/d*V* along all step edges. This is consistent with the Rashba edge states developed in Bi_2_Se_3_ due to the strong SOC.^[^ ^17^ ^]^ Figure 1d shows a large‐scale topographic image of the film annealed in UHV condition, which displays similar atomic morphology of 1 QL high islands with identical step height compared to the functionalized film in Figure 1b. However, the d*I*/d*V* maps at *V_B_* = −0.5 V show a striking difference (Figure 1e), where the contrast in d*I*/d*V* associated with the Rashba edge states has disappeared. The observation demonstrates that the edge states are sensitive to the Se content at the edge termination. Since all the STM measurements were performed at room temperature, our observation shows that the energy scale of Rashba edge states is large enough for switching them on and off in ambient conditions.

**Figure 2:**
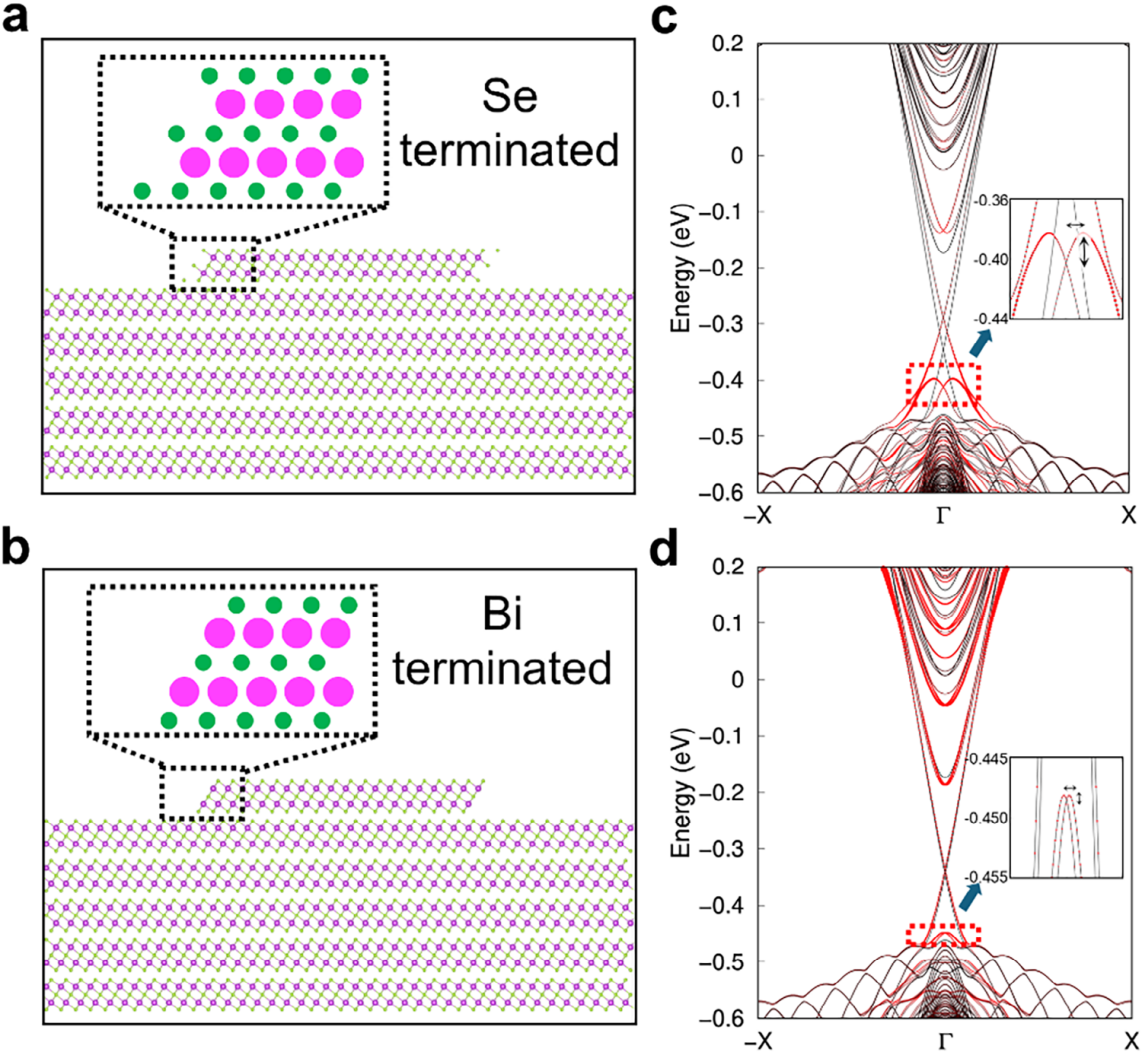
Theoretical electronic structures of Bi_2_Se_3_ films with different edge termination. a,b) Atomic structure of a 5 QL film with step edges terminated by Se and Bi atoms that represents Se functionalized and defunctionalized edges, respectively. c,d) Band structure of (a,b), respectively. The red color is proportional to the band projection at the step edge. The red dotted boxes and zoomed insets mark the energy and momentum range where Rashba edge states appear, which show large enhancement of Rashba splitting for the Se‐terminated case compared to the Bi‐terminated one.

To investigate how Se functionalization affects the edge states, we studied the electronic structures using the tight‐binding model based on DFT for a 5 QL Bi_2_Se_3_ film with a pair of step edges on top. The step edges can be terminated in several different atomic configurations depending on the Se content (Figure S1, Supporting Information).^[^ ^20^ ^]^ **Figure** **2**a,b shows atomic structures with the most Se rich case and Se poor case, and Figure 2c,d shows their band structure, respectively. The bulk band and topological surface states exist for both edge terminations with a similar shape. However, the Rashba edge states below the Dirac point (red boxes in Figure 2c,d) change drastically with the edge termination. The Se‐terminated edge shows an additional band confined to the edge with large Rashba splitting. Previous work showed that this band is spin‐polarized with the spin direction perpendicular to the edge with some out of plane components,^[^ ^17^ ^]^ which corroborates Rashba splitting of the band. Meanwhile, the Bi‐terminated edge has those bands shifted toward the valence bulk band and much smaller Rashba splitting. In general, the appearance of the Rashba band and Rashba splitting increases with the amount of Se at the edge due to the enhanced electric potential gradient that induces inversion symmetry breaking between the top and bottom QL (Figure S1, Supporting Information). Quantitatively, the Rashba strength *α_R_* and Rashba splitting energy *E_R_* of edge states increase with the Se composition at the edge, from *α_R_* = 0.233 eV·Å/(2π) and *E_R_* = 0.7 meV for the most Se‐poor edge, to *α_R_* = 1.395 eV·Å/(2π) and *E_R_* = 15 meV for the most Se‐rich edge (Table S1, Supporting Information), which substantiates tunability of Rashba parameters by edge functionalization. We note that the increased electric potential gradient by Se termination also induces 60‐meV split between the Dirac points of top and bottom topological surface states in Figure 2c, which is much smaller for the Bi‐terminated case in Figure 2d. We also note that there are Rashba states appear at the energy range of bulk conduction band (*E* > −0.2 eV), but they are not edge states but surface states delocalized over the whole terraces and cannot be observed as d*I*/d*V* localized along the step edges.^[^ ^3^, ^4^ ^]^ The theoretical results are consistent with the experiment where Se‐functionalized edges display a strong edge state signal while defunctionalized edges do not.

**Figure 3:**
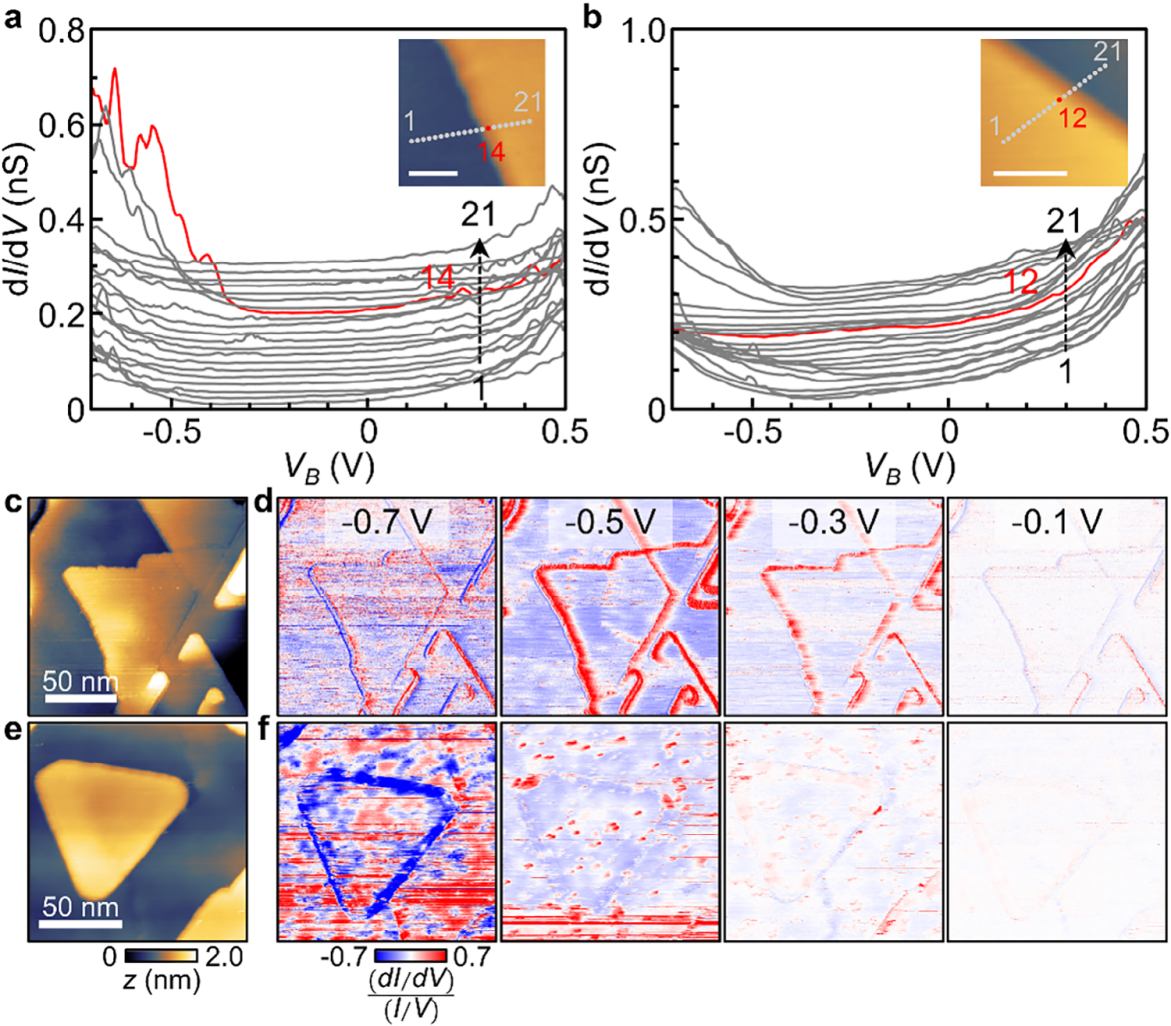
d*I*/d*V* spectroscopy at the Se functionalized and defunctionalized step edges. a,b) d*I*/d*V* spectra across the step edges functionalized and defunctionalized by Se atoms, respectively. The d*I*/d*V* curves are offset for clarity. c) Topographic image of Bi_2_Se_3_ film with Se functionalized edges. d) d*I*/d*V* maps for *V_B_* = −0.7–−0.1 V taken at the same area of (c). e) Topographic image of Bi_2_Se_3_ film with Se defunctionalized edges. f) d*I*/d*V* maps for *V_B_* = −0.7–−0.1 V taken at the same area of (e).

The d*I*/d*V* spectroscopy was taken to measure the LDOS and compare it with the band structures in Figure 2. **Figure** **3**a,b shows d*I*/d*V* spectra taken across the step edges that are functionalized and defunctionalized, respectively. For both cases, the spectra inside the bulk (grey lines) show a typical curve with the minimum around *V_B_* = −0.35 V, which indicates that Bi_2_Se_3_ films possess topological surface states with the Dirac point *E_D_* = −0.35 eV regardless of the Se functionalization.^[^ ^15^ ^]^ However, the spectra right on top of the step edges are drastically different depending on the functionalization. The spectra across the Se functionalized edge show a large increase in d*I*/d*V* for *eV_B_* ≤ *E_D_* (Figure 3a), which indicates the existence of localized Rashba edge states as predicted in Figure 2b. However, the defunctionalized edge shows no increase but slight depression in d*I*/d*V* for *eV_B_* ≤ *E_D_* (Figure 3b). The observation is consistent with the band calculation that shows Rashba edge states pushed into the bulk valence band (Figure 2d). The d*I*/d*V* maps confirm that such change in spectroscopy generally applies to all step edges (Figure 3c–f). Figure 3c,d shows the topographic image and d*I*/d*V* maps of the film annealed in Se flux. When *V_B_* = −0.5 and −0.3 V, the d*I*/d*V* signal significantly increases along the step edges due to the Rashba edge states. In contrast, the film annealed in UHV shows a drastic change in edge signal. The topographic image still displays step edges with the same height (Figure 3e), but d*I*/d*V* maps did not show any increase in the signal along the step edges but slight depression for *V_B_* = −0.5 V (Figure 3f). The result demonstrates that the topological surface states remain the same, but the edge states are modified by Se functionalization.

**Figure 4:**
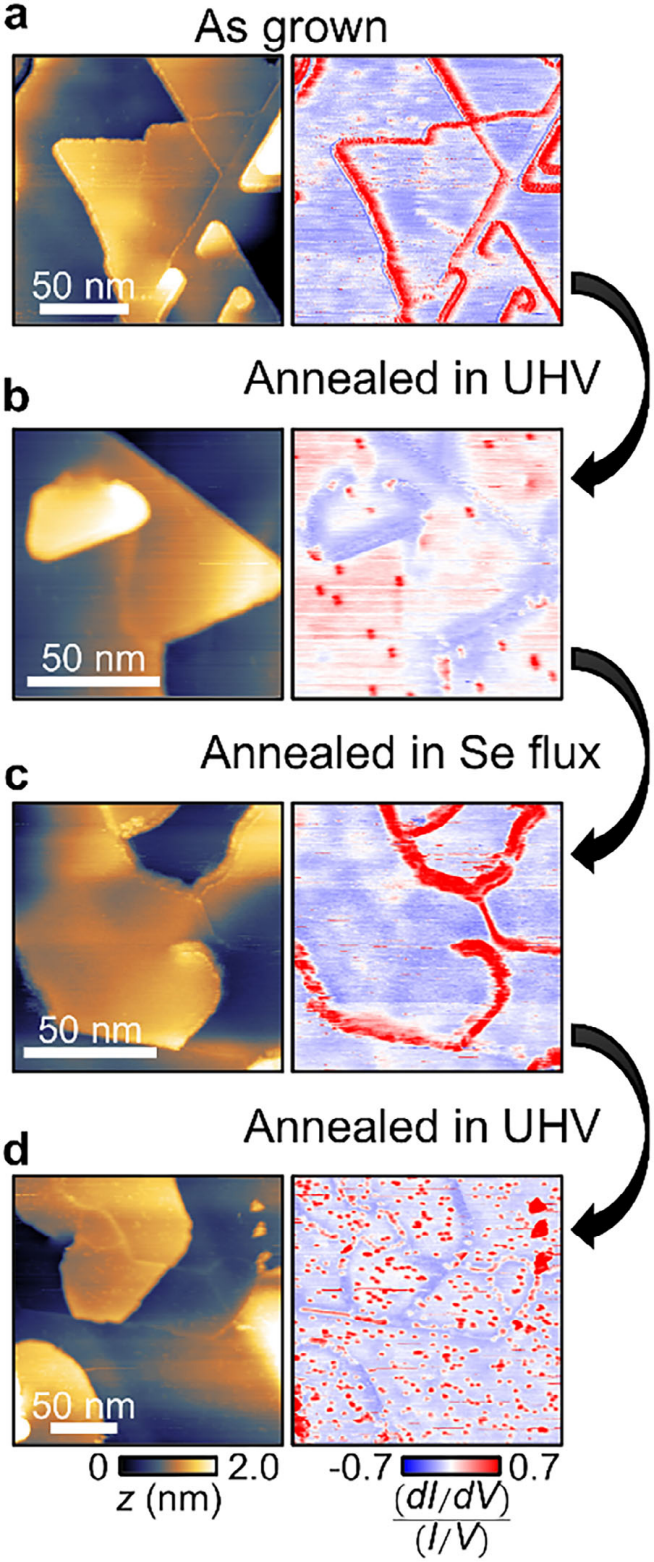
Reversible modification of Rashba edge states by Se functionalization. Topographic images and d*I*/d*V* maps for a) the as‐grown film, b) after annealing in UHV, c) after annealing in Se flux, and d) after second annealing in UHV.

Finally, we confirm Se functionalization and defunctionalization are reversible by alternatively annealing in Se flux and UHV (**Figure** **4**). The d*I*/d*V* maps were taken by STM after each step at room temperature. The as‐grown film displays Se functionalized edges with Rashba edge states due to the excessive Se flux during the growth (Figure 4a). Then, annealing the film in UHV defunctionalizes the step edges and removes the Rashba edge states (Figure 4b). These defunctionalized edges can be functionalized again by annealing in a Se flux environment, which clearly recovers the edge state signal in the d*I*/d*V* map (Figure 4c). Again, the second annealing in UHV defunctionalizes the edges and removes the Rashba edge states (Figure 4d). We note that d*I*/d*V* maps in Figure 4b,c displays increased number of red dots, which are atomic Se vacancies that induce defect states around the Dirac point.^[^ ^21^ ^]^ Increased number of Se vacancies are consistent with the removal of Se atoms from the surface by UHV annealing. In the current study, the goal was to confirm the reversibility, so we did not test beyond two cycles. However, STM images display no significant degradation in the films after each cycle, so we speculate the process can be repeated many more times. The reversibility of the functionalization at room temperature proves that it is an effective tool to switch on and off the Rashba edge states in ambient conditions.

## 3 Conclusion

In summary, we show that the emergent Rashba edge states that arise at the step edges of Bi_2_Se_3_ can be controlled at room temperature by functionalization and defunctionalization of atomic termination. The functionalization and defunctionalization are achieved by terminating step edges with Se atoms or reducing and changing the termination with Bi, which is experimentally performed by annealing in situ in MBE with or without Se flux. This effect is confirmed by using in situ STM and DFT calculations, which both highlight that the Rashba edge states are present only for functionalized edges but suppressed for defunctionalized ones. Moreover, DFT calculation suggests that the fundamental driver for this effect is the strength of the SOC dependent on the atomic configuration of the termination, which effectively tunes the energy scale of the Rashba effect at the step edges. Importantly, the process is shown to be fully reversible, i.e., step edges can be functionalized and defunctionalized in turn to repeatedly switch the Rashba edge states on and off. This work highlights the importance of the atomic configuration and detailed geometry of step edges as a subtle tuning knob for local electric potential gradient as well as the effective Rashba interaction strength. While our results do not guarantee the realization of spintronic devices operating at room temperature from these Rashba edge states, proof‐of‐principle demonstration of reversible switching of spin texture significantly increases a feasibility of these films for device applications. Further development on growth technique and transport characterization is required for realizing practical devices. For example, the switching of Rashba edge states could be further tailored by creating 1D wires at steps utilizing surface science knowledge, which could be deployed to integrate other quantum materials that can add additional functionality to these novel edge states, such as superconductors or magnets, or as a route to create a self‐aligned 1D gate metal localized at the step boundary.^[^ ^22^, ^23^ ^]^ The Fermi level should be tuned to the energy range of Rashba edge states, which can be realized by Sb substitution^[^ ^24^ ^]^ or electrical gating of the films.^[^ ^25^ ^]^ For further fabrication processes, the Bi_2_Se_3_ films should be capped with proper materials to protect them from the degradation by air exposure.^[^ ^26^ ^]^ Lastly, our work implies that local electric potential gradient tunes the SOC, and we expect that the same effect can be achieved ex situ through an electronic means such as ionic gating and electronic gating with a self‐aligned gate structure applied directly to the step edge.^[^ ^27^, ^28^ ^]^ The gating will greatly improve the energy efficiency by consuming several orders of magnitude less energy per switching than annealing process.

## 4 Experimental Section

### Sample Growth of Bi_2_Se_3_ Thin Films

The samples were grown in a home‐built molecular beam epitaxy (MBE) reactor at Oak Ridge National Laboratory, which was in situ coupled to the STM. The Bi_2_Se_3_ samples were grown on Al_2_O_3_ substrates, which were mounted ex situ on the sample holder using silver paste. The substrates were cured at ≈150 °C, then cooled and exposed to UV‐generated ozone for 10 min to clean the surface. The substrates were pumped down and transferred into the MBE reactor, then heated to ≈600 °C and exposed to a flux of Se. They were then cooled to ≈135 °C where 3 QL of Bi_2_Se_3_ were deposited. The film was then heated to 235 °C where the remainder of the film was grown, and then cooled to room temperature and transferred into the STM. The growth was controlled by the amount of Bi, which was calibrated prior to the growth to a flux of 2 × 10^13^ cm^−2^ s^−1^, and Se was calibrated to about ten times of the Bi flux.

### Scanning Tunneling Microscopy/Spectroscopy

The STM/STS was taken by Omicron VT‐STM operated at ultrahigh vacuum (<10^−9^ torr) and room temperature. The thin film samples grown in the separate MBE chamber were transferred to the STM chamber through the directly connected UHV radial distribution chamber to avoid any exposure to the air. The d*I/*d*V* spectra were taken by a conventional lock‐in technique with a modulation voltage of 50 mV and a modulation frequency of 1 kHz. The d*I/*d*V* maps were taken in closed‐loop mode, where the surface was scanned at a certain bias with the current feedback on while the lock‐in modulation voltage was applied to measure d*I/*d*V* simultaneously.

### Theoretical Calculations

To investigate the electronic properties of the various terminated terrace structures, we used the Slater−Koster type tight‐binding (TB) model using hopping parameters in the previous study,^[^ ^17^ ^]^ which successfully reproduces the DFT band structure for the Bi_2_Se_3_ bulk near the Fermi level. Here, three *p* orbitals were assumed for each of the Bi and Se atoms.

## Conflict of Interest

The authors declare no conflict of interest.

## Supporting information



Supporting Information

## Data Availability

The data that support the findings of this study are available from the corresponding author upon reasonable request.

## References

[1] M. Z. Hasan , C. L. Kane , Rev. Mod. Phys. 2010, 82, 3045.

[2] X. L. Qi , S. C. Zhang , Rev. Mod. Phys. 2011, 83, 1057.

[3] P. D. C. King , R. C. Hatch , M. Bianchi , R. Ovsyannikov , C. Lupulescu , G. Landolt , B. Slomski , J. H. Dil , D. Guan , J. L. Mi , E. D. L. Rienks , J. Fink , A. Lindblad , S. Svensson , S. Bao , G. Balakrishnan , B. B. Iversen , J. Osterwalder , W. Eberhardt , F. Baumberger , P. Hofmann , Phys. Rev. Lett. 2011, 107, 096802. 21929260 10.1103/PhysRevLett.107.096802

[4] Z. H. Zhu , G. Levy , B. Ludbrook , C. N. Veenstra , J. A. Rosen , R. Comin , D. Wong , P. Dosanjh , A. Ubaldini , P. Syers , N. P. Butch , J. Paglione , I. S. Elfimov , A. Damascelli , Phys. Rev. Lett. 2011, 107, 186405. 22107654 10.1103/PhysRevLett.107.186405

[5] J. C. R. Sánchez , L. Vila , G. Desfonds , S. Gambarelli , J. P. Attané , J. M. De Teresa , C. Magén , A. Fert , Nat. Commun. 2013, 4, 2944. 24343336 10.1038/ncomms3944

[6] Y. Ando , T. Hamasaki , T. Kurokawa , K. Ichiba , F. Yang , M. Novak , S. Sasaki , K. Segawa , Y. Ando , M. Shiraishi , Nano Lett. 2014, 14, 6226. 25330016 10.1021/nl502546c

[7] C. H. Li , O. M. J. Van't Erve , J. T. Robinson , Y. Liu , L. Li , B. T. Jonker , Nat. Nanotechnol. 2014, 9, 218. 24561354 10.1038/nnano.2014.16

[8] K. Kondou , R. Yoshimi , A. Tsukazaki , Y. Fukuma , J. Matsuno , K. S. Takahashi , M. Kawasaki , Y. Tokura , Y. Otani , Nat. Phys. 2016, 12, 1027.

[9] J. C. Rojas‐Sánchez , S. Oyarzún , Y. Fu , A. Marty , C. Vergnaud , S. Gambarelli , L. Vila , M. Jamet , Y. Ohtsubo , A. Taleb‐Ibrahimi , P. Le Fèvre , F. Bertran , N. Reyren , J. M. George , A. Fert , Phys. Rev. Lett. 2016, 116, 096602. 26991190 10.1103/PhysRevLett.116.096602

[10] Y. Wang , D. Zhu , Y. Wu , Y. Yang , J. Yu , R. Ramaswamy , R. Mishra , S. Shi , M. Elyasi , K.‐L. Teo , Y. Wu , H. Yang , Nat. Commun. 2017, 8, 1364. 29118331 10.1038/s41467-017-01583-4 PMC5677620

[11] S. M. Hus , X. G. Zhang , G. D. Nguyen , W. Ko , A. P. Baddorf , Y. P. Chen , A.‐P. Li , Phys. Rev. Lett. 2017, 119, 137202. 29341679 10.1103/PhysRevLett.119.137202

[12] W. Ko , G. D. Nguyen , H. Kim , J. S. Kim , X. G. Zhang , A.‐P. Li , Phys. Rev. Lett. 2018, 121, 176801. 30411944 10.1103/PhysRevLett.121.176801

[13] M. Brahlek , Adv. Mater. 2020, 32, 2005698. 10.1002/adma.202005698 33145882

[14] S.‐Y. Xu , Y. Xia , L. A. Wray , S. Jia , F. Meier , J. H. Dil , J. Osterwalder , B. Slomski , A. Bansil , H. Lin , R. J. Cava , M. Z. Hasan , Science 2011, 332, 560. 21454752 10.1126/science.1201607

[15] Y. Xia , D. Qian , D. Hsieh , L. Wray , A. Pal , H. Lin , A. Bansil , D. Grauer , Y. S. Hor , R. J. Cava , M. Z. Hasan , Nat. Phys. 2009, 5, 398. 10.1103/PhysRevLett.103.146401 19905585

[16] H. J. Zhang , C. X. Liu , X. L. Qi , X. Dai , Z. Fang , S. C. Zhang , Nat. Phys. 2009, 5, 438.

[17] W. Ko , S.‐H. Kang , J. Lapano , H. Chang , J. Teeter , H. Jeon , Q. Lu , A.‐H. Chen , M. Brahlek , M. Yoon , R. G. Moore , A.‐P. Li , ACS Nano 2024, 18, 18405. 38970487 10.1021/acsnano.4c02926

[18] N. I. Fedotov , S. V. Zaitsev‐Zotov , Phys. Rev. B 2017, 95, 155403.

[19] Y. Xu , G. Jiang , J. Chiu , L. Miao , E. Kotta , Y. Zhang , R. R. Biswas , L. A. Wray , New J. Phys. 2018, 20, 073014.

[20] N. S. Virk , PhD Thesis, EPFL, Lausanne 2016.

[21] Z. Alpichshev , R. R. Biswas , A. V. Balatsky , J. G. Analytis , J. H. Chu , I. R. Fisher , A. Kapitulnik , Phys. Rev. Lett. 2012, 108, 206402. 23003161 10.1103/PhysRevLett.108.206402

[22] B. Jäck , Y. Xie , B. Andrei Bernevig , A. Yazdani , Proc. Natl. Acad. Sci. USA 2020, 117, 16214. 32601184 10.1073/pnas.2005071117 PMC7368272

[23] B. Jäck , Y. Xie , J. Li , S. Jeon , B. A. Bernevig , A. Yazdani , Science 2019, 364, 1255. 31196882 10.1126/science.aax1444

[24] T. Arakane , T. Sato , S. Souma , K. Kosaka , K. Nakayama , M. Komatsu , T. Takahashi , Z. Ren , K. Segawa , Y. Ando , Nat. Commun. 2012, 3, 636. 22273674 10.1038/ncomms1639

[25] D. Kim , S. Cho , N. P. Butch , P. Syers , K. Kirshenbaum , S. Adam , J. Paglione , M. S. Fuhrer , Nat. Phys. 2012, 8, 459.

[26] N. Koirala , M. Brahlek , M. Salehi , L. Wu , J. Dai , J. Waugh , T. Nummy , M.‐G. Han , J. Moon , Y. Zhu , D. Dessau , W. Wu , N. P. Armitage , S. Oh , Nano Lett. 2015, 15, 8245. 26583739 10.1021/acs.nanolett.5b03770

[27] D. S. Fartab , J. Guimarães , M. Schmidt , H. Zhang , Phys. Rev. B 2023, 108, 115305.

[28] D. Shcherbakov , P. Stepanov , S. Memaran , Y. Wang , Y. Xin , J. Yang , K. Wei , R. Baumbach , W. Zheng , K. Watanabe , T. Taniguchi , M. Bockrath , D. Smirnov , T. Siegrist , W. Windl , L. Balicas , C. N. Lau , Sci. Adv. 2021, 7, abe2892. 10.1126/sciadv.abe2892 PMC7846175 33514554

