# Supplementary material for: Reversible Modification of Rashba States in Topological Insulators at Room Temperature by Edge Functionalization
Source: Adv Sci (Weinh). 2025 Nov 20;13(4):e19814. doi: 10.1002/advs.202519814 (PMC12822435; doi:10.1002/advs.202519814)
Supplement: Supplementary file 1 — Supporting Information [file ADVS-13-e19814-s001.docx]

*Supporting Information of*

**Reversible Modification of Rashba States in Topological Insulators at Room Temperature by Edge Functionalization**

Wonhee Ko^1†*^, Seoung-Hun Kang^2,3,4,5†^, Qiangsheng Lu^2^, An-Hsi Chen^2^, Gyula Eres^2^, Ho Nyung Lee^2^, Young-Kyun Kwon^3,4^, Robert G. Moore^2^, Mina Yoon^2*^, Matthew Brahlek^2*^

^1^*Department of Physics and Astronomy, The University of Tennessee, Knoxville, Tennessee 37996, USA*

^2^*Materials Science and Technology Division, Oak Ridge National Laboratory, Oak Ridge, Tennessee 37831, USA*

*^3^Department of Information Display, Kyung Hee University, Seoul 02447, Korea*

*^4^Department of Physics, and Research Institute for Basic Sciences, Kyung Hee University, Seoul 02447, Korea*

*^5^Research Center for Technology Commercialization, Korea Institute of Science and Technology Information (KISTI), Seoul 02456, Korea*

^†^These authors contributed equally to this work

Email: [wko@utk.edu](mailto:wko@utk.edu), [myoon@ornl.gov](mailto:myoon@ornl.gov), [brahlekm@ornl.gov](mailto:brahlekm@ornl.gov)

**1. The 1D Rashba edge states based on the edge shape of the nanoribbon on 5 QL Bi_2_Se_3_**

We have examined all stable Bi_2_Se_3_ edge structures reported in the literature.^[1]^ As shown in Fig. 3.2 of the cited ref. ^[1]^, the energetics of various Bi_2_Se_3_ edges depend on the chemical potentials of Se and Bi. Among these edges, only II_Se_($\frac{\mathbf{4}\sqrt{\mathbf{3}}}{\mathbf{9}}$ Bi) has an armchair configuration, while the others exhibit different types of zigzag configurations. Regardless of edge type, our study discovered that a 1D Rashba edge state is generated in all structures. The strength of the 1D Rashba edge state varies with the Se chemical potential, as summarized in Table. R2. Under the Se-rich condition of our MBE growth, our calculations indicate that the stable edges - IV_Se_(-2 Bi), III_se_(-1Bi, our model structure), and II_Se_($\frac{\mathbf{4}\sqrt{\mathbf{3}}}{\mathbf{9}}$ Bi) - exhibit strong 1D Rashba edge states. This finding is consistent with our STM/STS measurement.


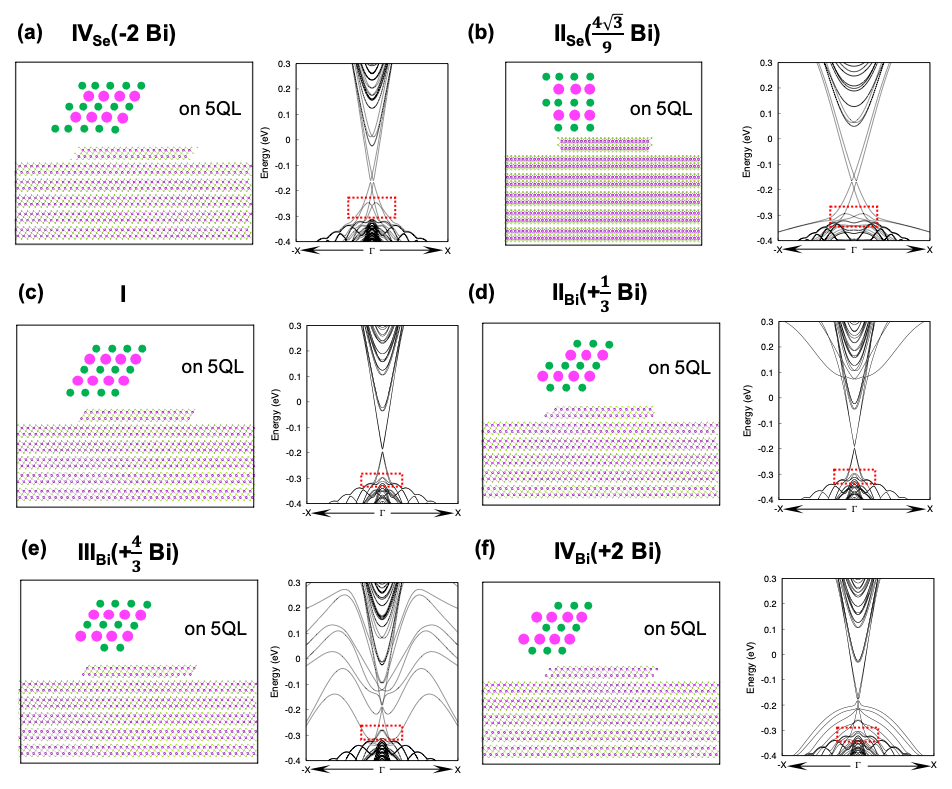


**Figure S1. The edge shape dependence of 1D Rashba states in nanoribbon on 5 QL Bi_2_Se_3_.** The structures of stable Bi_2_Se_3_ nanoribbons were analyzed as a function of the Se chemical potential, and their edge shapes were determined. Six different structures, ranging from the most Se-rich to Se-poor conditions, were identified and labeled as (a) to (f).^[1]^ The red dotted boxes mark the energy and momentum range where Rashba edge states appear, which show greater Rashba splitting for more Se on the edge.

**Table S1. The Rashba strength *α_R_* and Rashba splitting energy *E_R_* vary for stable edges with different shapes, depending on the Se chemical potential**. The values of *α_R_* and *E_R_* are evaluated by fitting band structures in Figure S1 to the equation $E^{\pm}\left( \vec{k} \right)=-\frac{\hbar^{2}k^{2}}{2m^{*}}\pm\alpha_{R}\left| \vec{k} \right|$, where $\alpha_{R}=2{E_{R}}/{k_{0}}$.

| Se-rich ¬ μ_Se_ (chemical potential of Se) ® Se-poor | | | | | | | |
| --- | --- | --- | --- | --- | --- | --- | --- |
| Stable  edge | IV_Se_(-2 Bi) | III_Se_(-1 Bi) | II_Se_($\frac{\mathbf{4}\sqrt{\mathbf{3}}}{\mathbf{9}}$ Bi) | I | II_Bi_(+$\frac{1}{3}$ Bi) | III_Bi_(+$\frac{4}{3}$ Bi) | IV_Bi_(+2 Bi) |
| *α_R_*  (eV* Å /2π) | 1.395 | 1.173 | 0.641 | 0.182 | 0.333 | 0.275 | 0.233 |
| *E_R_* (meV) | 15 | 11.3 | 10 | 0.4 | 1 | 0.7 | 0.7 |

**2. Schematic of charge-spin conversion device using Rashba edge states**

**
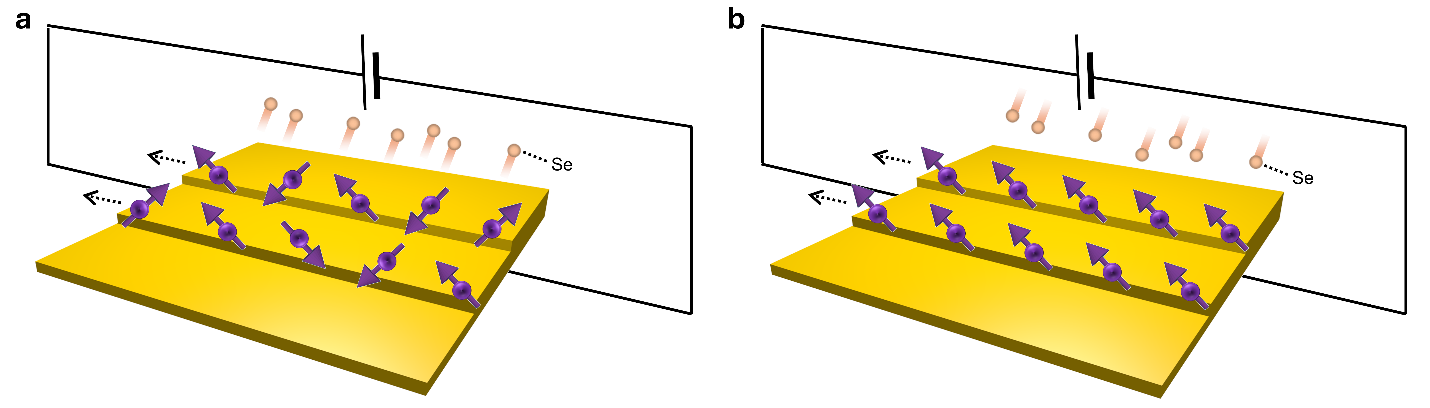
**

**Figure S2. Schematic of a charge-spin conversion device made of Bi_2_Se_3_ films with aligned terraces and step edges.** Bias is applied so the current flows along the step edges. When the step edges are defunctionalized (a), the current does not induce a spin polarization, whereas when the step edges are Se functionalized (b), the current through the Rashba edge states induces a spin polarization.

**References**

[1] N. S. Virk, Vol. PhD, Lausanne, EPFL, 2016.
